# 阿扎胞苷联合来那度胺治疗伴TP53突变骨髓增生异常肿瘤患者临床研究

**DOI:** 10.3760/cma.j.cn121090-20250930-00454

**Published:** 2025-11

**Authors:** 欣 严, 澄豪 郭, 婵 杨, 承棋 林, 丹丹 宋, 志梅 蔡, 莹 王, 莲 王, 峥 葛

**Affiliations:** 1 东南大学附属中大医院血液科，东南大学血液病研究所，南京 210009 Department of Hematology, Zhongda Hospital, School of Medicine, Southeast University, Institute of Hematology Southeast University, Nanjing 210009, China; 2 徐州医科大学附属连云港医院（连云港市第一人民医院），连云港 222000 The Affiliated Lianyungang Hospital of Xuzhou Medical University / The First People's Hospital of Lianyungang, Lianyungang 222000, China; 3 盱眙人民医院，盱眙 211700 Xuyi People's Hospital, Xuyi 211700, China

**Keywords:** 阿扎胞苷, 来那度胺, 骨髓增生异常肿瘤, TP53突变, 治疗疗效, Azacitidine, Lenalidomide, Myelodysplastic neoplasm, TP53 mutation, Treatment outcome

## Abstract

**目的:**

探索应用阿扎胞苷联合来那度胺治疗伴TP53突变的骨髓增生异常肿瘤（myelodysplastic neoplasms, MDS）患者的有效性和安全性；初步探索治疗有效的潜在机制。

**方法:**

纳入2021年1月至2025年6月于东南大学附属中大医院就诊并接受阿扎胞苷联合来那度胺治疗的伴TP53突变的16例MDS患者，评估疗效和安全性；分析TP53突变与治疗反应的相关性；通过转录组测序和生物信息学分析技术初步探索治疗有效相关分子标志物。

**结果:**

16例MDS患者中男性8例，女性8例，中位年龄69.5（52～82）岁，分子国际预后积分系统（molecular international prognostic scoring system, IPSS-M）中低危1例、高危2例、极高危13例。16例TP53突变患者中双打击突变11例，单打击突变5例。9例患者治疗有效，总体有效率（Overall response rate, ORR）为56.3％，复合完全缓解率（Composite complete remission，CRc）为31.3％（5/16），血液学改善率为25.0％（4/16）（其中贫血改善1例，贫血及血小板减少改善2例，中性粒细胞减少改善1例）。治疗有效组患者治疗后TP53突变中位等位基因突变频率（Variant allele frequency, VAF）显著低于治疗前［16.5％（0～83.9％）对65.6％（20.4％～93.4％），*P*＝0.017］。复杂核型伴TP53突变治疗有效率为53.8％（7/13），且57.1％（4/7）治疗后复杂核型消失。最常见的3/4级非血液学不良反应为感染（9/16，56.3％），包括肺炎（4/16，25.0％）、消化道感染（3/16，18.8％）、肛周感染（1/16，6.3％）和血流感染（1/16，6.3％）。初步机制研究显示，TP53患者应用阿扎胞苷联合来那度胺治疗有效可能与CBX8基因高表达相关。

**结论:**

阿扎胞苷联合来那度胺可有效治疗伴TP53突变MDS患者，安全性和耐受性良好。在治疗有效患者中可显著降低TP53突变VAF。治疗前CBX8基因高表达可能与TP53突变患者治疗起效相关。

## Abstract (en)

**Objective:**

To assess the efficacy and safety of azacitidine combined with lenalidomide in MDS patients and explore potential mechanisms of therapeutic response.

**Methods:**

Sixteen MDS patients with TP53 mutations received azacitidine plus lenalidomide at ZhongDa Hospital, Southeast University（January 2021–June 2025）. Efficacy and safety were assessed, and TP53 mutation status was correlated with treatment response. Whole-transcriptome sequencing and bioinformatics were used to explore molecular biomarkers associated with therapeutic efficacy.

**Results:**

Sixteen patients（median age 69.5 years, range 52–82; 8 males, 8 females）were enrolled. According to the Molecular International Prognostic Scoring System（IPSS-M）, 1, 2, and 13 patients were classified as median low, high, and very high risk, respectively. Among 16 TP53-mutated patients, 11 had biallelic mutations and 5 had monoallelic mutations. Overall response rate was 56.3％（9/16）, composite complete remission rate（CRc）was 31.3％（5/16）, and hematology improvement rate was 25％（4/16）. Among TP53-mutated patients, the response rate was 56.3％（9/16）, with variant allele frequency dropping from 65.6％ to 16.5％ in responders（*P*＝0.017）. In patients with TP53 mutations and complex karyotype, response rate was 53.8％（7/13）, with 57.1％（4/7）showing disappearance of CK post-treatment. The most common grade 3–4 nonhematologic adverse events were infections（9/16, 56.3％）, including pneumonia（4/16, 25.0％）, gastrointestinal infections（3/16, 18.8％）, perianal infections（1/16, 6.3％）and sepsis（1/16, 6.3％）. High CBX8 expression may be linked to treatment response.

**Conclusion:**

Azacitidine plus lenalidomide is an effective and safe therapy for MDS, including patients with TP53 mutations and complex karyotypes. Treatment markedly reduces TP53 variant allele frequency in responders, and high CBX8 expression may predict therapeutic response.

骨髓增生异常肿瘤（Myelodysplastic neoplasms, MDS）是一种克隆性造血干细胞恶性疾病，其特征是血细胞减少、髓系细胞一系或多系发育异常、无效造血，高风险演变为急性髓系白血病（Acute myeloid leukemia, AML）[1]–[3]。MDS患者在临床表现及疾病进展（PD）上具有显著异质性，强调个体化治疗[3]–[6]。对于高危MDS患者，主要治疗目标是延缓潜在的AML进展。去甲基化药物包括阿扎胞苷（Azacitidine, AZA）和地西他滨，广泛用于中高危MDS治疗，有效率可达40％～60％，但对于伴有复杂核型和TP53突变的患者疗效差[7]–[13]。目前，伴有TP53突变的MDS患者尚无有效治疗方案，即使接受移植的患者预后仍不良。因此探索针对TP53突变的新治疗方案具有重要临床意义。

来那度胺（Lenalidomide, LEN）是一种免疫调节性药物，最早用于治疗伴有5号染色体短臂缺失（5q−）的低危MDS[14]–[15]。既往研究表明，AZA+LEN治疗高危MDS患者疗效优于单药治疗，且多数患者可耐受，具有良好的有效性和安全性[16]–[21]，但在伴有TP53突变的MDS患者中疗效和治疗前后TP53基因突变动态变化情况尚不明确。本研究旨在探索在伴TP53突变的MDS患者中使用AZA+LEN治疗的疗效和治疗前后基因突变动态变化情况，并探索治疗起效的初步机制，报道如下。

## 病例与方法

一、病例

本研究为回顾性队列研究。纳入2021年1月至2025年6月于东南大学附属中大医院就诊的MDS患者，纳入标准：①根据WHO2022诊断标准诊断为MDS，且骨髓/外周血原始细胞比例<20％，伴TP53突变；②年龄18周岁以上；③肝肾功能、心功能正常，美国东部肿瘤协作组（ECOG）评分<2分；④自愿签署知情同意书。排除标准：①由已存在骨髓增殖性肿瘤、骨髓增殖性肿瘤/骨髓增生异常肿瘤演变而来的MDS；②治疗前6个月内发生任何威胁生命的出血事件（颅内出血），或存在严重溃疡、胃肠穿孔、腹瘘、胃肠梗阻病史；③治疗前4周内发生严重感染，受到严重外伤或接受外科重大手术；④存在重度免疫缺陷或HIV抗体检测阳性，活动性乙型肝炎、丙型肝炎、结核病。筛选流程见图1。本研究通过东南大学附属中大医院伦理委员会批准后实施（批件号：2020ZDSYLL170-P01）。

**图1:**
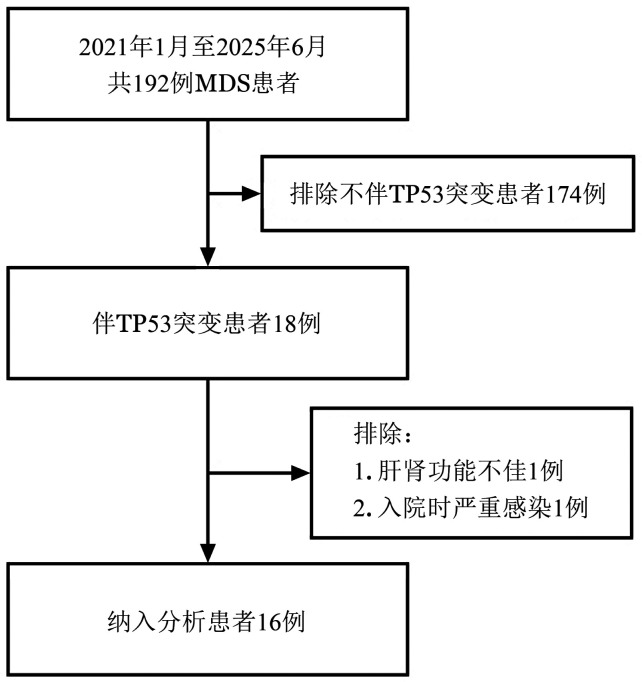
阿扎胞苷联合来那度胺治疗伴TP53突变骨髓增生异常肿瘤（MDS）患者纳入流程图

二、治疗方案

阿扎胞苷75 mg·m^−2^·d^−1^，皮下注射，第1～7天；来那度胺10 mg/d，口服，第1～21天，根据血常规结果调整剂量、频率、疗程，如若ANC低于1.0×10^9^/L和（或）PLT<50×10^9^/L，来那度胺减量为5 mg/d并可进一步减量为5 mg隔日1次直至血常规恢复正常。若治疗期间HGB<70 g/L，予输血支持治疗。28 d为1个疗程。

三、疗效评估标准

MDS疗效判定采用IWG-2023疗效标准[22]，以治疗期间达到的最佳疗效为主要分析目标，总有效率（Overall response rate，ORR）定义为完全缓解（Complete remission，CR）、等同完全缓解（Complete remission equivalent, CR_equivalent_）、完全缓解伴血常规两系恢复（Complete remission bilineage, CR_bi_）、完全缓解伴血常规一系恢复（Complete remission unilineage, CR_uni_）、完全缓解伴部分血常规恢复（Complete remission with partial hematologic recovery, CRh）、部分缓解（Partial remission, PR）及血液学改善（Hematology improvement, HI）率之和。复合缓解率（Composite Complete remission rate, CRc）定义为CR、CR_equivalent_、CR_bi_、CR_uni_和CRh之和。

四、基因突变检测与分析标准

取患者骨髓并分离单个核细胞，常规提取DNA且制备DNA全基因组文库。使用PCR引物扩增目的基因组（涵盖58个血液肿瘤相关基因），将目标区域DNA富集后，采用Illumina Hiseq测序平台进行测序。等位基因突变频率（Variant allele frequency, VAF）≥2％的基因突变纳入分析，所有检测出的外显子区通过千人基因组计划（1000 Genomes Project）、COSMIC及PolyPhen-2数据库筛选出致病基因。具体方法详见文献[23]–[24]。

五、转录组测序方法与分析标准

取患者骨髓并分离单个核细胞，提取RNA并检测RNA降解程度以及是否有污染，Nanodrop检测RNA的纯度；Qubit对RNA浓度进行精确定量；去除rRNA，将RNA打断成250～300 bp的短片段，以短片段RNA为模板，加入六碱基随机引物合成cDNA，纯化双链DNA后进行PCR富集得到特异性cDNA文库，构建完成后对文库进行库检，库检合格后进行Illumina测序。具体方法详见文献[25]。

六、生信分析方法与标准

采用fastqc和fastp对下机数据进行质控清洁获得clean reads，采用hisat2包将clean reads与比对到人类参考基因组GRCh38进行比对，然后利用featureCounts包获取每个样本中比对到参考基因组上的Reads Counts。采用FPKM（Fragment Per Kilobase of transcript, per Million mapped reads）对Counts矩阵进行标准化。利用edgeR软件包分析两组样本的差异表达基因，按照log_2_FC绝对值大于2、FDR<0.05条件筛选显著差异表达基因。采用韦恩图和热图展示显著差异基因在不同样本中的表达情况。

七、随访

随访截至2025年7月31日，随访资料来源于住院/门诊病历及电话随访记录。治疗30 d内死亡定义为早期死亡。患者总生存（Overall survival, OS）时间按初次治疗时间起至死亡或接受异基因造血干细胞移植（Allogeneic hematopoietic stem cell transplantation, allo-HSCT）或随访截止日期计算。患者无白血病生存（Leukemia free survival, LFS）时间按初次治疗时间起至发生白血病转化或接受allo-HSCT或死亡日期计算。

八、统计学处理

采用SPSS 24.0软件进行统计分析，采用R 4.1.2和Graphpad 8.0绘制图形。计量资料符合偏态分布，以中位数（范围）表示，治疗前后TP53突变频率比较采用配对秩和检验。生存分析采用Kaplan-Meier法。双侧*P*<0.05为差异有统计学意义。

## 结果

一、基线临床与分子生物学特征

16例患者中男8例，女8例，中位年龄69.5（52～82）岁，依据WHO2022诊断标准：MDS伴低原始细胞（MDS-LB）2例、MDS伴TP53双等位突变（MDS-biTP53）11例、MDS伴原始细胞增多Ⅰ型（MDS-IB1）1例、MDS伴原始细胞增多Ⅱ型（MDS-IB2）2例。中位HGB 67.5（43～126）g/L，中位WBC 3.18（1.15～34.83）×10^9^/L，中位ANC 1.16（0.15～32.49）×10^9^/L，中位PLT 56（6～324）×10^9^/L，中位外周血原始细胞比例0％（0％～7％），中位骨髓原始细胞比例3.8％（0％～13.0％）。15例患者染色体核型异常（93.75％），其中复杂染色体核型13例（86.7％）。修订版国际预后积分系统（Revised international prognostic score system, IPSS-R）细胞遗传学分组：预后良好1例，预后中等1例，预后差1例，预后极差13例。分子国际预后积分系统（Molecular international prognostic scoring system, IPSS-M）预后分组：中低危1例，高危2例，极高危13例（表1）。16例TP53突变患者治疗前基因突变检测结果见图2，其中单打击TP53突变5例，双打击TP53突变11例；TP53突变患者伴随突变较少，仅检出TET2（2/16，12.5％）、EZH2（1/16，6.25％）、SF3B1（1/16，6.25％）、U2AF1（1/16，6.25％）、NOTCH1（1/16，6.25％）、DNMT3A（1/16，6.25％）、GATA2（1/16，6.25％）突变。

**表1:** 阿扎胞苷联合来那度胺治疗伴TP53突变骨髓增生异常肿瘤（MDS）患者临床特征与治疗疗效

例号	诊断	染色体核型	基因突变情况	IPSS-M	HGB（g/L）	WBC（×10^9^/L）	PLT（×10^9^/L）	疗效评估	起效疗程
1	MDS-biTP53	复杂核型	TP53	极高危	102	7.58	18	CR_equivalent_	2
2	MDS-biTP53	复杂核型	TP53	极高危	66	1.22	27	CR	3
3	MDS-biTP53	复杂核型	TP53	极高危	85	2.36	68	CR	2
4	MDS-biTP53	复杂核型	TP53	极高危	61	2.11	72	HI	2
5	MDS-LB	46,XX[20]	TP53	中低危	126	3.97	108	HI	2
6	MDS-biTP53	46,XY,add（3）（p11）[20]	TP53、EZH2、NOTCH1	高危	83	22.42	199	HI	2
7	MDS-IB2	复杂核型	TP53、DNMT3A、TET2	极高危	75	1.44	168	CRh	1
8	MDS-biTP53	复杂核型	TP53、GATA2	极高危	73	8.58	157	CRh	2
9	MDS-LB	复杂核型	TP53	极高危	55	2.38	44	HI	1
10	MDS-biTP53	46,XX,del（7q）[20]	TP53	极高危	63	34.83	324	PD	-
11	MDS-biTP53	复杂核型	TP53、U2AF1	极高危	60	5.46	136	PD	-
12	MDS-IB1	复杂核型	TP53、TET2	高危	84	9.10	13	PD	-
13	MDS-biTP53	复杂核型	TP53	极高危	46	5.22	36	PD	-
14	MDS-IB2	复杂核型	TP53	极高危	57	2.23	10	PD	-
15	MDS-biTP53	复杂核型	TP53、SF3B1	极高危	43	2.24	6	PD	-
16	MDS-biTP53	复杂核型	TP53	极高危	69	1.15	17	PD	-

**注** MDS-biTP53：MDS伴TP53双等位突变；MDS-LB：MDS伴低原始细胞；MDS-IB：MDS伴原始细胞增多；IPSS-M：分子国际预后积分系统；CR：完全缓解；CR_equivalent_：等同完全缓解；CRh：完全缓解伴部分血常规恢复；HI：血液学改善；PD：疾病进展；-：未获得疗效

**图2:**
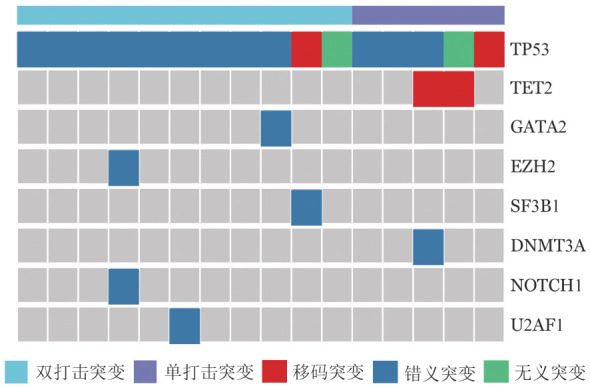
接受阿扎胞苷联合来那度胺治疗伴TP53突变骨髓增生异常肿瘤患者基因突变分布情况

二、疗效、预后与不良反应

AZA+LEN中位治疗2（1～11）个疗程。16例患者中9例治疗有效，ORR为56.3％（9/16），CRc为31.3％（5/16）（其中CR 2例、CR_equivalent_ 1例、CRh 2例），HI率为25％（4/16）（其中贫血改善1例，贫血、血小板减少改善2例，中性粒细胞减少改善1例）；复杂核型伴TP53突变治疗有效率为53.8％（7/13），且4例（57.1％，4/7）治疗后复杂核型消失。治疗起效患者临床与分子生物学特征见表1。IPSS-M预后分组较低危组（中低危）治疗有效1例（1/1，100％），较高危组（中高危、高危、极高危）治疗有效8例（8/15, 53.3％）；治疗起效中位疗程为2（1～3）个，其中第一疗程起效2例（22.2％），第二疗程起效6例（66.7％），第三疗程起效1例（11.1％）。

中位随访9（1.5～37）个月，16例患者中11例死亡，5例进展为AML，1例在经过6个疗程AZA+LEN治疗后接受allo-HSCT治疗。中位OS时间为9（95％*CI*：2～16）个月，中位LFS时间为8（95％*CI*：4～12）个月。

本研究中患者发生的不良反应见表2。最常见的3/4级血液学不良反应为贫血（10/16，62.5％）；最常见的3/4级非血液学不良反应为感染（9/16，56.3％），包括肺炎（4/16，25.0％）、消化道感染（3/16，18.7％）、肛周感染（1/16，6.3％）和血流感染（1/16，6.3％）治疗期间没有患者发生治疗相关早期死亡；3例患者因治疗后肺部感染而死亡，1例患者治疗1个疗程后因严重血细胞减少而终止AZA+LEN治疗，换用其他方案治疗。

**表2:** 阿扎胞苷联合来那度胺治疗伴TP53突变骨髓增生异常肿瘤过程中不良反应［例（％）］

不良反应	1～2级	3～4级
贫血	2（12.5）	10（62.5）
中性粒细胞减少	1（6.3）	8（50.0）
血小板减少	1（6.3）	9（56.3）
肺炎	0（0）	4（25.0）
血流感染	0（0）	1（6.3）
消化道感染	2（12.5）	3（18.8）
肛周感染	0（0）	1（6.3）
上呼吸道感染	1（6.3）	0（0）
恶心呕吐	1（6.3）	0（0）
便秘	2（12.5）	0（0）
腹泻	1（6.3）	0（0）
皮疹	1（6.3）	0（0）

三、治疗前后TP53等基因突变动态变化情况

本研究中11例（11/16，68.8％）患者于治疗前后行基因突变检测，患者治疗前后基因突变动态变化情况见图3。治疗有效组患者TP53突变中位VAF治疗后显著低于治疗前［65.6％（20.4％～93.4％）对16.5％（0％～83.9％），*P*＝0.017］。

**图3:**
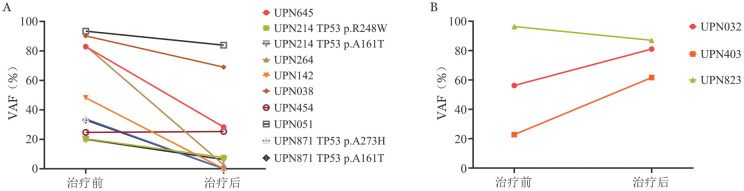
阿扎胞苷联合来那度胺治疗伴TP53突变骨髓增生异常肿瘤治疗前后TP53等位基因突变频率（VAF）变化情况 **A** 治疗有效组；**B** 治疗无效组 g003

四、TP53突变患者治疗有效机制初步探索

本研究中16例治疗前有TP53突变的患者中9例患者治疗有效，其中双打击6例（CR 2例，CR_equivalent_ 1例，CRh 1例，HI 2例），单打击3例（疗效达CRh 1例，HI 2例），7例患者治疗无效。RNA-seq测序结果显示，治疗有效患者治疗前CBX8基因高表达，而治疗后该基因表达显著下降；治疗无效患者治疗前该基因低表达，治疗后该基因表达上升，提示该基因与治疗起效相关。治疗有效组患者治疗后相较于治疗前免疫调控相关基因（CCL20、NCAM1、THEMIS等）表达显著上调（图4）。

**图4:**
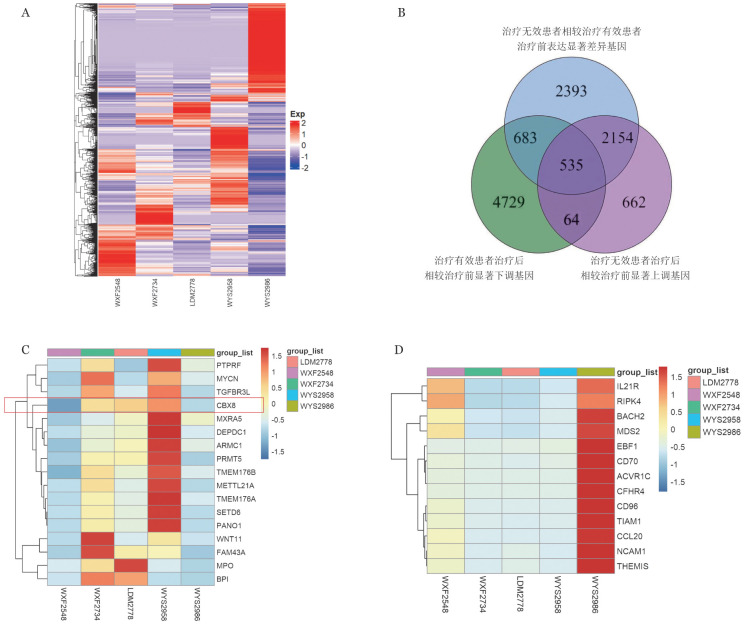
TP53突变骨髓增生异常肿瘤患者治疗起效分子机制初步探索 **A** AZA+LEN治疗有效组与治疗无效组治疗前后样本基因表达差异比较；**B** 差异表达基因筛选流程；**C** AZA+LEN治疗有效组治疗前高表达基因差异比较；**D** AZA+LEN治疗有效组治疗后高表达基因差异比较 **注** AZA：阿扎胞苷；LEN：来那度胺

## 讨论

MDS中高危患者，尤其是伴双等位TP53突变的MDS患者预后较差，目前无有效治疗方案。去甲基化药物治疗已在既往临床应用中显现出较好疗效，被批准用于治疗IPSS-R中危及以上危险分层患者，但对伴TP53突变和伴复杂核型MDS患者治疗疗效较差，因此有必要探索针对TP53突变的更为有效的治疗方案，以更好指导临床实践。

既往研究报道单用AZA治疗MDS患者ORR为24％～67.8％[16],[26]–[28]，但对伴TP53突变和伴复杂核型的高危MDS患者疗效不佳，分别为8.7％和7.9％[28]，而本研究中AZA+LEN治疗在伴TP53突变MDS患者中ORR分别为56.3％，且在治疗有效患者中可显著降低TP53突变VAF并使复杂核型消失。提示AZA+LEN治疗对伴TP53突变和伴复杂核型的MDS患者较单用AZA治疗具有更好的疗效，推荐该类患者尝试采用AZA+LEN治疗。Sekeres等[16]研究显示，AZA+LEN治疗起效患者皆在6个治疗疗程内起效；其他研究也表明，治疗起效患者中位治疗至起效时间为3～3.7个月，即多数患者在治疗3个疗程内起效[15],[17]。本研究中患者常在治疗前3个疗程起效，提示治疗有效的患者多数在3个疗程内起效，治疗3个疗程后未起效出现PD，可考虑更换治疗方案；若治疗有效或无明显证据表明PD的患者，应继续应用AZA+LEN方案治疗。本研究我们分析AZA+LEN治疗期间的不良反应发现，血细胞减少及相关感染是最主要不良反应，其他非血液学不良反应发生率低，与文献[14]–[19]报道一致。进一步证实了AZA+LEN治疗的安全性，多数患者可耐受。

既往研究证实，TP53突变是MDS患者预后不良和发生白血病转化（Leukemic transformation, LT）的危险因素，且TP53突变对OS和LT的影响与TP53突变类型和VAF大小相关[7]–[11]。我们既往研究[25]亦发现TP53突变VAF在PD和LT过程中有显著增高，初次测序时有TP53突变并不一定会使MDS患者发生PD/LT，只有病程中新增/克隆扩增的TP53突变才会促使MDS患者发生PD/LT，若治疗能使TP53突变VAF降低，则患者不易发生PD/LT；提示如果能降低TP53突变VAF，抑制TP53突变恶性克隆扩增，可能可以阻止MDS患者发生PD/LT。本研究中治疗有效MDS患者TP53突变VAF皆有下降，提示AZA+LEN治疗可能可以抑制TP53恶性克隆扩增，阻止MDS患者发生PD/LT。

RNA-seq检测结果提示治疗有效的TP53突变患者相较于治疗无效患者，治疗前CBX8基因高表达，而治疗后显著下降；CBX8基因是重要表观调控基因，主要功能是调控组蛋白去甲基化，调控细胞分化与细胞周期，是一种原癌基因。CBX8可直接结合TP53蛋白或其靶基因启动子区域，抑制TP53的转录活性，从而促进肿瘤细胞增殖；并可通过与去乙酰化酶SIRT1协同作用，抑制p53的乙酰化修饰，进一步降低其稳定性和活性，从而抑制凋亡信号通路。既往研究表明CBX8在AML发病中起关键作用[29]–[30]。因而我们推测CBX8基因高表达可能是TP53突变患者对去甲基化药物治疗敏感的标志，未来可能可以作为筛选对去甲基化药物治疗有效的TP53突变患者的标志，但仍需大样本数据进一步证实。治疗有效组患者治疗后免疫调控相关基因表达显著增高，提示AZA+LEN可能通过激活免疫调控相关基因，增强免疫细胞功能以杀伤肿瘤细胞促使治疗起效，但仍需进一步研究。未来有望在大样本患者群体中开展针对伴TP53突变的MDS患者前瞻性AZA+LEN治疗临床试验，以进一步明确治疗疗效并探索治疗起效分子机制。

总之，本研究证实了AZA+LEN对伴有TP53突变和复杂核型患者疗效显著，且治疗有效患者TP53突变VAF显著降低。治疗前CBX8基因高表达可能与TP53突变患者治疗有效相关。因本研究为单中心研究，纳入临床研究患者数量较少，有待多中心、大样本量的前瞻性临床研究进一步证实。
